# Polyglutamic acid grafted dopamine modified collagen-polyvinyl alcohol hydrogel for a potential wound dressing

**DOI:** 10.1080/15685551.2021.1984007

**Published:** 2021-09-28

**Authors:** Guofei Yu, Changkai Yang, Nianhua Dan, Weihua Dan, Yining Chen

**Affiliations:** a National Engineering Research Center of Clean Technology in Leather Industry, Sichuan University, Chengdu, Sichuan, China; b Research Center of Biomedical Engineering, Sichuan University, Chengdu, Sichuan, China

**Keywords:** Collagen pga hydrogel water-absorbing

## Abstract

Natural collagen has good biocompatibility and ability to promote tissue regeneration and repair, but the poor mechanical properties and intolerance of degradation of natural collagen limit its applications in the biomedical field. In this research, we synthesized a skin wound repair hydrogel with good biological activity, high strength and excellent water absorption properties. Inspired by the theory of wet healing, dopamine was introduced into the side chain of the water-absorbing polymer polyglutamic acid to synthesize a cross-linking agent (PGAD) with both water absorption and cell adhesion ablities, and then it was introduced into collagen/polyvinyl alcohol (PVA-COL) system to form a double network hydrogel. Scanning electron microscope observation of the morphological characteristics of the hydrogel showed that after the introduction of PGAD, the hydrogel formed an obvious pore structure, and the swelling rate showed that the introduction of PGAD significantly improved the water absorption rate of the hydrogel.In addition, PVA-COL-PGAD hydrogel has good mechanical properties and water absorption behavior.In vitro experimental results revealed that the hydrogel has good biocompatibility. In vivo wound healing experiments showed that hydrogel can promote wound healing process.These results indicated that our hydrogel has great potential as a medical wound dressing.

## Introduction

Hydrogel is a polymer cross-linked network that contains a large amount of water. Due to its high compatibility with wet healing theory, it has great advantages in the field of wound repair. So far, scientists have used the theory of dissipative toughening mechanism to construct complex structures to improve the problem of insufficient mechanical properties of hydrogels, such as interpenetrating networks, double networks, and nanoparticle cross-linked networks. However, most flexible hydrogels are affected by water swelling, resulting in a decrease in mechanical properties and toughness, and their applications range is limited. Therefore, it is a challenging subject to develop a balanced swelling hydrogel with excellent mechanical strength and multiple functions [1–3].

Collagen (COL) is the most abundant protein in mammals. Because of its good biocompatibility, inducing tissue regeneration, and biodegradability, it is an ideal raw material for tissue repair [4]. Collagen hydrogel has the incomparable advantages of other traditional hydrogels. It can not only give a moist environment for wound healing, accelerate the formation of epithelium, but also play a role in coagulation and hemostasis. At the same time, it releases angiogenesis-promoting morphogens such as growth factors and ECM fragments during the degradation process, which can promote the formation of burr blood vessels and help wound healing [5,6]. However, the problems of poor mechanical properties and rapid degradation of hydrogels formed by pure collagen have limited its applications in biomedical materials. Therefore, researchers combined collagen with other polymer materials to improve the insufficient mechanical properties of collagen hydrogels during wound healing [7–9].

Polyvinyl alcohol (PVA) is a water-soluble polymer material. PVA hydrogel is widely used in biomedical fields such as wound dressing and cartilage repair because of its good biocompatibility [10]. The pure PVA hydrogel has neither biological activity nor good hydrophilicity, and it has big defects in the applications of wound dressings. The medical material developed by compounding PVA with natural polymer materials with good biological activity, biocompatibility and biodegradability is a kind of wound dressing with great potential [11–13]. The hydrogel prepared by compounding COL and PVA has not only good mechanical properties of PVA, but also a compound hydrogel dressing with excellent biological activity [14]. However, the mere introduction of collagen does not significantly promote the swelling balance of the hydrogel, and cannot improve the local dehydration and drying problems that may exist in the PVA hydrogel during use. Therefore, it is particularly important to find a new material or method to improve this defect.

Polyglutamicacid (γ-PGA) is a water-soluble polymer formed by the polymerization of D-glutamic acid and L-glutamic acid through γ-glutamine bonds. There are a large number of free carboxyl groups in the side chain of its molecular structure [15]. It has excellent moisture retention, high viscosity, biocompatibility and biodegradability. Studies have shown that the large number of free carboxyl groups contained in the γ-PGA structure enables the γ-PGA hydrogel to swell thousands of times its own weight in water, and has extremely superior water absorption properties [16,17]. At the same time, its polymer properties make it possible to form a three-dimensional network structure in an aqueous solution, with excellent water-locking properties. In addition, studies have shown that the combination of dopamine (DA) and other compounds containing catechol groups with the material can improve the biocompatibility of the material [18,19].

In this study, our goal was to develop a wound dressing hydrogel with biological activity, high strength and excellent water absorption. By grafting dopamine onto the side chain of polyglutamic acid, a new type of macromolecular crosslinking agent PGAD was prepared and applied to polyvinylalcohol-collagen (PVA-COL) double network structure to improve the water absorption properties of polyvinyl alcoholhydrogels.The chemical structure, surface and internal structure, rheological properties, mechanical properties and swelling properties of the obtained PVA-COL-PGAD hydrogel were studied. Cell compatibility was evaluated by methyl thiazolidazole tetrazolium(MTT)assay, and finally in vivo cell compatibility was evaluated by full-thickness skin injury model.Subsequently, the wound healing effect of hydrogels in vivo on full-thickness skin defects was also evaluated to investigate the in vivo cytocompatibility.

## Experimental

### Materials

Collagen (COL) from porcine skin was prepared according to our published study [20].The molecular weight of COL was approximately 30 kDA. Polyglutamicacid (γ-PGA,molecular weight, MW ~ 1,000,000) and dopamine hydrochloride (DA, 98%) were purchased from Sigma-Aldrich (St.Louis,MO,USA). 1-Ethy-3-(3-dimethylaminopropyl-carbodiimide)hydrochloride (EDC·HCl), N-hydroxysuccinimide (NHS) and 2-(N-morpholino) ethanesulfonic acid (MES) were purchased from Aladdin Reagent Inc (Shanghai). Fetal bovine serum (FBS), penicillin–streptomycin solution, and 3-(4,5-dimethylthiazol-2-yl)-2,5-diphenyltetrazolium bromide (MTT) assay were purchased from HyClone (Logan, UT, USA). Other chemicals and reagents were purchased from Bioneec Biological Technical Limited Company (Chengdu, China). Unless noted, chemicals and reagents were used without further purification.

### Prepration of crosslinking agent PGAD

Refer to the method of Wei Chen et al. and make slight changes [21]. 3 g γ-PGA was dissolved in 150 ml MES buffer (pH = 5.2 ± 0.2) to prepare a 2% solution. At room temperature, add the above solution according the mass ratio of EDC/NHS of 1:1 (2.69 g), and activate it with magnetic stirring for 30 min. After the above steps are completed, adding 0.74 g DA makes the molar mass ratio of DA to γ-PGA is 0.5:1. Under normal temperature conditions, pass nitrogen protection, avoid light and magnetically stir the reaction for 24 h. After the reaction was completed, use a dialysis bag with a molecular weight cut-off of 3500 Da to dialysis for 3 days in the dark, and frequently change the deionized water. After freeze-drying, polyglutamic acid grafted with dopamine can be obtained, which is recorded as PGAD.

### Prepration of PVA-COL-PGAD hydrogel

The prepration process of PVA-COL-PGAD is shown in Figure 1.10 g polyvinyl alcohol (PVA) was dissolved in 100 g deionized water under 90°C water bath to obtain 10% PVA aqueous solution.A 10 mg/ml collagen solution was prepraed by dissolving collagen in an aqueous solution of acetic acid (0.1 mol /L) and the pH was adjusted to 7.4 (NaOH).Then, a certain quality of PGAD was dissolved in deionized water and prepared into 0 g/ml, 0.05 g/ml, 0.1 g/ml, 0.2 g/ml PGAD solution for use.Thereafter, 2.5 ml collagen solution and 5 ml PVA solution was mixed and stirred 4 h at 4°C.Finnally,1 ml of PGAD of different concentrations was added to form a precursor solution, and finally, freezed at −20°C for 12 hours and thawed at room temperature for 12 hours to prepare the hydrogel.The obtained hydrogels were maked as PVA-COL,PVA-COL-1,PVA-COL-2,PVA-COL-3,respectively,according to the degree of PGAD.

**Figure 1.**
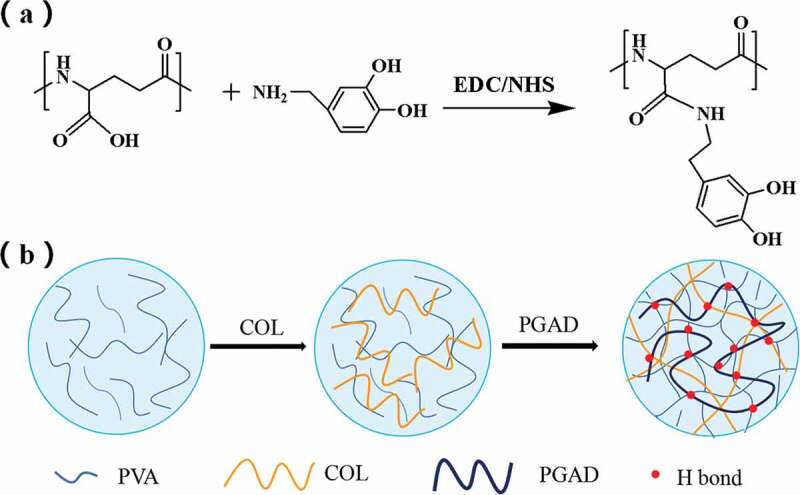
Graft reaction of γ-PGA with DA (a), The schematic diagram of PVA-COL-PGAD hydrogel formation (b)

### Chemical structure of PGAD

The structural analysis of PGAD was performed using a Fourier transform infrared (FTIR) spectrometer (Spectrum One, PerkinElmer, Inc., Waltham, MA). The samples were triturated with potassium bromide (KBr) in the ratio of 1 : 100 (mg /mg) and then compressed into flake. All spectra were collected in transmission mode at 4 cm^−1^ resolution ratio and recorded in the wavelength range of 4000–400 cm^−1^. The measurements were carried out in a dry atmosphere at room temperature.

The ^1^H NMR spectra of PGA and PGAD were recorded on a nuclear magnetic resonance spectrometer (Bruker AVII-400 MHz) using D_2_O as a solvent. 1–3 mg freeze-dried samples were put into a tube and then 0.55 mL D_2_O was injected.

### FT-IR analysis of hydrogels

The PVA, PVA-COL and PVA-COL-3 hydrogel that have been frozen and thawed once are dried with a freeze dryer. The specimens mixed with KBr were pressed into tablets with ratios of 1 to 100–150. Fourier transform infrared (FTIR) spectra were measured from the tablets using a FTIR spectrophotometer (Nicolet iS10, Thermo Scientific CO, America). The scanning condition is: the transmission mode is collected under the wavelength range of 4000 ~ 500 cm^−1^, and the resolution is 4 cm^−1^. The measurements were carried out in a dry atmosphere at room temperature.

### Thermogravimetric analysis

Approximately 3–5 mg of hydrogels were pressed into a cylindrical holder and thermogravimetric (TG) analysis performed on a thermal analyzer (Netzsch TG 209, Germany) from 30 to 600 °C at a heating rate of 10 K/min. All measurements in the instrument were conducted under an atmosphere of nitrogen.

### Differential scanning calorimetry and X-ray diffraction analysis

Put each group of hydrogel samples on the X-ray diffraction table, with the smoother surface as the test surface, and the scanning angle is 5–50°.

Cut the freeze-dried hydrogel material into small pieces of appropriate size, accurately weigh 2 ~ 4 mg. Place the sample in a DSC aluminum crucible, seal it and perform DSC testing. The testing parameters are: 20 mL/min nitrogen protection, temperature range 25 ~ 250 °C, the heating rate is 10 K/min, and the blank crucible is used as a reference.

### Phase morphology of dried hydrogels

The surface and cross-sectional microscopic morphology of the freeze-dried hydrogel were observed under a scanning electron microscope (Japan, Hitachi, SEM). Before observation, a gold layer was sprayed on the surface of the hydrogel.

### Rheological tests

A Rheometric Scientific HAAKE (MARS, German) strain control rheometer equipped with a 20 mm parallel plate was used to characterize the dynamic rheological properties of the hydrogel at room temperature. The hydrogel is loaded into the 1.5 mm gap between the plates and allowed to relax until the normal force is zero. The dynamic frequency sweep is performed at an angular velocity of 0.1 Hz to 100 Hz with a strain amplitude of 1.0%.

### Water content tests

In order to observe the polyglutamic acid content of grafted dopamine and the influence of different freeze-thaw times on the water content of the prepared hydrogels, the water content of each group of hydrogel samples of 1, 2, and 3 freeze-thaw cycles was measured. The surface moisture of each group of samples to be tested is removed through filter paper and weighed (W_1_), then freeze-dried in a freeze dryer to a constant weight and weighed (W_2_). Three parallel tests were set for each group of samples. Calculate the average, and the results are expressed in terms of mean and variance.The water content(WC) of hydrogels were calculated by eqn (1).

_(1)_ $$WC(\%)=[(W_{1}-W_{2})/W_{1}]\times 100\%$$

### Water return rate tests

After measuring the water content of the hydrogel, the dried hydrogel was soaked in deionized water for 24 hours until the water absorption balance, and then use filter paper to absorb the surface water, accurately weigh the mass of the hydrogel after water absorption, and the water return rate was passed. The quality difference before and after the hydrogel absorbs water is determined.The water return rate(RSD) were calculated by _eqn (2_),

_(2)_ $$RSD(\%)=[(W_{3}-W_{2})/W_{2}]\times 100\%$$

where W2 is the dry weight of hydrogel and W3 is the weiight of hydrogel after adsorbing water.

### Evaluation of in vitro biocompatibility

L929 fibroblasts are used in the MTT (methyltetrazole) assay to evaluate the cytocompatibility of the hydrogel. The proliferation of cells cultured in the hydrogel extract was evaluated. In short, the sample was cut to size and sterilized by radiation. Next, the hydrogel was immersed in the cell culture medium at 37°C for 24 hours to obtain an extract. The cell culture medium is DMEM medium supplemented with 1% (v/v) antibiotics and 10% (v/v) calf serum. The cells (cell concentration 5 × 10 4 /ml) were cultured with 0.1ml extract in a humidified atmosphere containing 5% CO 2 and incubated at 37°C with fresh cell culture medium on 96-well plastic tissue culture plates . At the same time, fresh medium served as a blank control group. Cell viability was evaluated after 1, 3 and 5 days of culture. At each time point, 20 μl/well of 0.5 mg/ml MTT solution was added to the culture and incubated at 37°C for 4 hours to form formazan crystals. Subsequently, DMSO (200μl/well) was added to dissolve the formazan crystals, and the optical density (OD) at 492nm was measured with a microplate reader (model 550, Bio Rad Corp. USA) to evaluate the degree of cytotoxicity.

### Evaluation of in vivo biocompatibility

In animal studies, a full-thickness skin defect model was constructed using healthy adult Sprague-Dawley rats weighing approximately 300 g. The procedure is as follows: prepare 10% glyoxal as a hydrate as an anesthetic and inject it intraperitoneally in rats at a dose of 300 mg/Kg. After anesthesia, the surgical site was depilated, the posterior surgical site was prepared and fixed to a sterile operating table. An approximately full-size skin incision wound was created using an iodophore to sterilize the surgical site and its surroundings and a sterile scalpel to remove full-thickness skin in the bandage on both sides of the spine10 mm x 20 mm.After debridement with normal saline, PVA-COL-1 hydrogel was applied to the surface of the wound on the right side, and the other side was the control group. Then the wound was covered with sterile gauze, fixed with a skin stapler, and finally wrapped with iron sand net. After the operation, the animals were sent to a clean animal room for breeding, and three consecutive days of penicillin injection were performed to prevent infection. The rats were sacrificed 1, 2, 3, or 4 weeks after the operation, and samples were taken. According to the standard protocol, hematoxylin and eosin (H&E) staining is used for histological observation, and immunohistological staining is used to detect basic fibroblast growth factor (bFGF), vascular endothelial growth factor (VEGF) and platelet-derived growth The expression factor (PDG). In addition, all laboratory animals should be handled in accordance with the guidelines for the use and care of laboratory animals established by the National Institutes of Health. All procedures performed on animals were first approved by the Animal Care and Use Committee of Sichuan University.

## Results and discussion

### Structure analysis of PGAD

**Figure 2.**
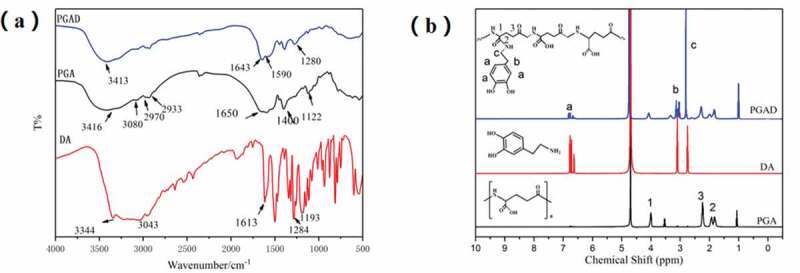
The FT-IR spectra analysis of DA, PGA and PGAD(a), ^1^H NMR spectra of DA, PGA and PGAD (b)

Figure 2a shows the FT-IR spectra of DA, PGAand PGAD. Compared with PGA, the weak absorption peaks at 1524 cm-1 and 1288 cm-1 can be designated as amide II band and amide III band absorption peaks, respectively, indicating that the amidation reaction occured between dopamine and PGA[21,22]. Also, the same result was obtained from the 1H NMR spectrum shown in Figure 2b. Compared with PGA, the chemical shifts at δ2.7, δ2.3, and δ6.3–6.8 ppm were assigned to the methylene hydrogen peak of dopamine, the methylene hydrogen peak adjacent to the amide bond, and phenyl proton peak in the PGA-DA chains,respectively.This indicates that dopamine was successfully introduced into the PGA side chain [23].

### FT-IR analysis of hydrogels

**Figure 3.**
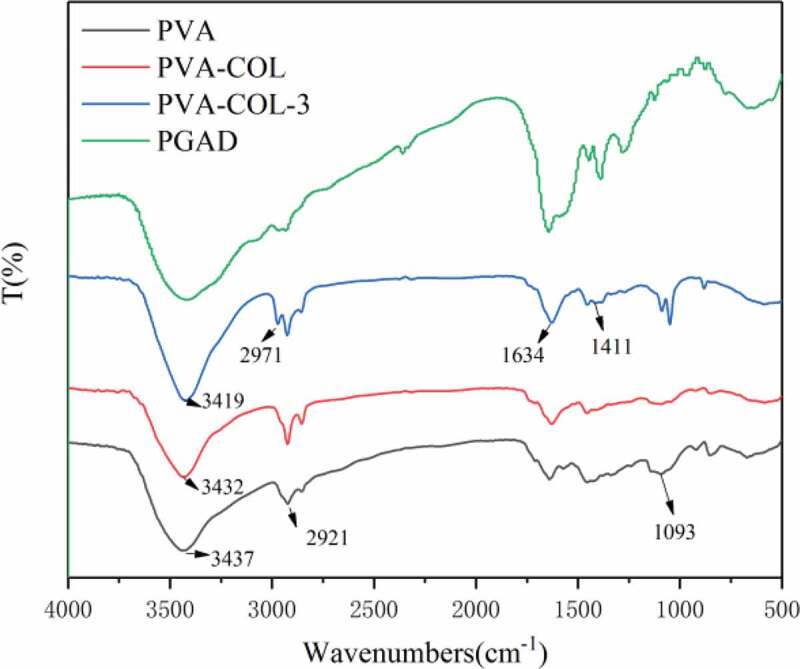
The FT-IR spectra analysis of PVA, PVA-COL and PVA-COL-3

Figure 3 shows the FT-IR of PVA, PVA-COL, and PVA-COL-3 hydrogels. It can be seen from the figure that the peak of PVA hydrogel at 3439 cm-1was attributed to the stretching vibration of O-H, and the peaks at 2923 cm-1 and 1094 cm-1were attributed to the stretching vibration of C-H and C-O, respectively [24],[25]

### Thermogravimetric analysis

**Figure 4.**
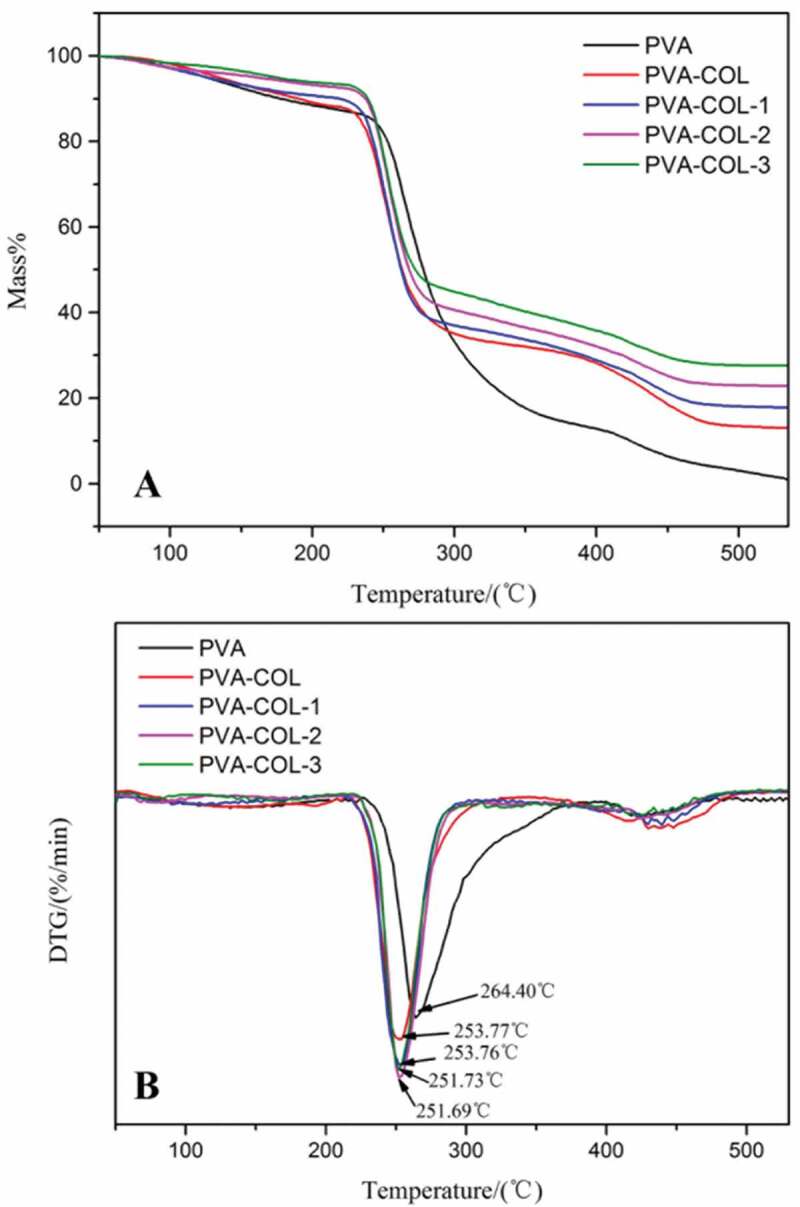
The TG and DTG curves of PVA, PVA-COL and PVA-COL-PGAD

Thermogravimetry is one of the commonly used methods to characterize the thermal stability of materials, which can reflect the interaction between the components of the hydrogel composite from the side. Figure 4(a) and 4(b) are the TG and DTG curves of PVA, PVA-COL, PVA-COL-1, PVA-COL-2, PVA-COL-3 hydrogel samples, respectively. It can be seen that there are roughly three weightless stages: the evaporation stage of free water and bound water, the breakdown of the cross-linking points inside the hydrogel and the decomposition of polymer molecules into small molecules of volatile substances, and the molecular lysis stage [26] Figure 4(b) that the temperature (Tm) at which the thermal weight loss rate of each group of hydrogels is the largest is between 250–255°C. Compared with PVA, the introduction of COL and different content of PGAD all lead to a decrease in the Tm value of the hydrogel. This may be due to the introduction of collagen destroys the physical cross-linking of the PVA molecules to a certain extent, which makes it easier to decompose into small molecular substancein the second stage of thermal decomposition. Molecular substance. However, the Tm values of the hydrogels with different dosages of PGAD and PVA-COL hydrogels are not much different, which shows that the introduction of PGAD has a weaker effect on the Tm values of PVA-COL hydrogels.

### Differential scanning calorimetry and X-ray diffraction analysis

**Figure 5.**
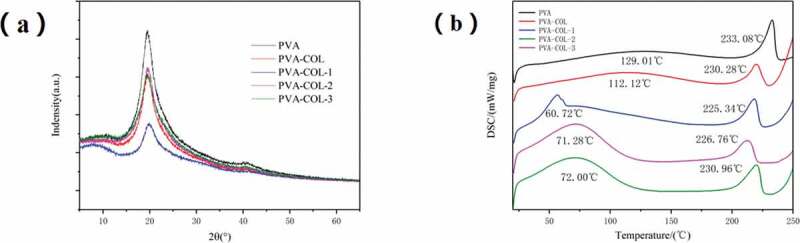
The XRD curves of hydrogels in each group(a), the DSC curves of hydrogels in each group(b)

PVA is a semi-crystalline polymer. It can be seen from Figure 5 that PVA shows three typical diffraction peaks at 2θ of 19.6°,22.9°,and 40.8°[27] Figure 5(b) is a graph of DSC test results of each group of hydrogels. It can be seen from the figure that the samples of each group have obvious absorption peaks at around 100 °C and around 230 °C. The absorption peak at about 100 °C is caused by the evaporation of water in the sample, and the absorption peak at about 230 °C is the melting absorption peak of PVA crystals. The introduction of collagen resulted in a slight decrease in the melt absorption peak, while the introduction of PGAD resulted in a significant decrease in the melt absorption peak. It shows that a strong hydrogen bond is generated between PGAD and PVA and COL, which inhibits the formation of PVA crystals, which is consistent with the results of XRD.

### Phase morphology of dried hydrogels

**Figure 6.**
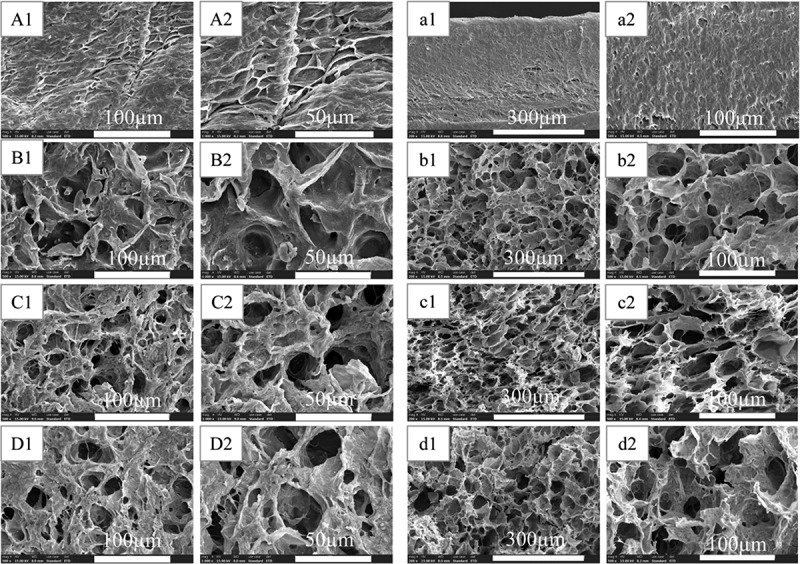
The SEM of lyophilized hydrogels (A,B,C,D surface morphology:500×,1000×;a,b,c,d cross-sectional morphology:200×,500×)

Scanning electron microscope was used to observe the microscopic morphology of each group of hydrogels, and the results are shown in Figure 6. Among them, A, B, C, and D are the surface microstructures of PVA-COL, PVA-COL-1, PVA-COL-2 and PVA-COL-3, respectively, and a, b, c, and d are the horizontal Cross-section microscopic morphology.

It can be seen from the surface topography of each group that there are filamentous substances in the surface topography of PVA-COL hydrogel, and the cross section is relatively smooth. After the introduction of PGAD, the filamentous material on the surface of the PVA-COL hydrogel became coarser, and obvious pore-like structures appeared. From the cross-sectional topography of each group of samples, it can be seen that the introduction of PGAD caused a large number of pores in the gel. Among them, the PVA-COL-1 and PVA-COL-2 hydrogels had a large number and uniform pore structure. This shows that the introduction of PGAD makes the PVA and COL macromolecules more closely linked, so that the hydrogel has thicker fibers with a more uniform structure, forming a more superior three-dimensional network structure. The pores in PVA-COL-3 are larger and smaller in number. This may be due to the higher PGAD content, which plays a role in filling between PVA-COL and reduces the existence of pore-like structures.In short, the chaotic surface and rough structure caused by the introduction of PGAD are conducive to cell adhesion and growth. The internal three-dimensional network structure and the existence of holes are also more conducive to the growth of cells and provide a good growth space for cells.

### Rheological analysis

**Figure 7.**
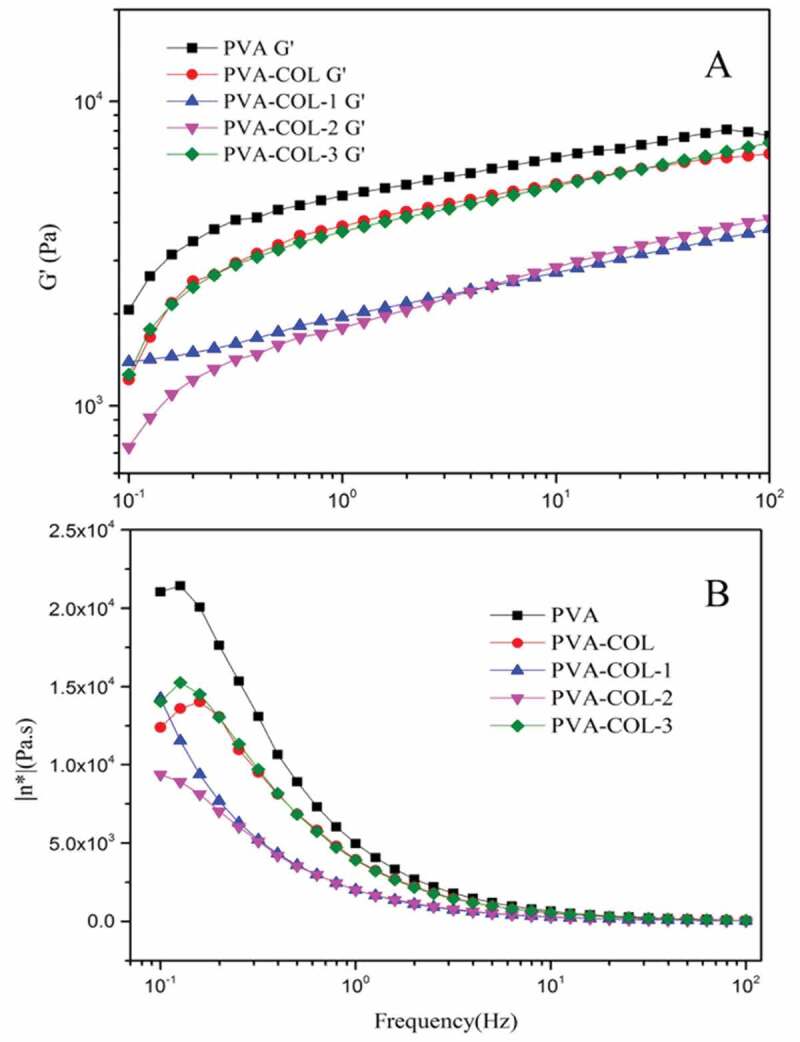
Characterization of rheological properties of hydrogels

Figure 7 shows the rheological properties of each group of hydrogels. The storage modulus G’ reflects the elasticity of the hydrogel material, and the loss modulus G” reflects the viscosity of the hydrogel material.It can be seen from Figure 7 that the G’ of each group of hydrogels increases with the increase of the shear frequency, and G” decreases with the increase of the shear frequency. This is the result of the viscoelasticity of the hydrogel [28],[29].

Comparing Figure 7(A) and (B), it can be found that the introduction of COL resulted in a decrease in the G’ and G” of the PVA hydrogel. After the introduction of PGAD, the G’ and G” of the PVA-COL hydrogel decreased first and then increased with the increase of PGAD content. The G’and G” of the PVA-COL-1 hydrogel were both critical point of data change after the introduction of PGAD content. This may be because the introduction of COL and a small amount of PGAD would hinder the formation of hydrogen bonds between PVA molecular chains. As the content of PGAD in the hydrogel increases, the three-dimensional structure of PGAD as a macromolecular substance makes the overall three-dimensional network structure of the hydrogel stronger, resulting in the rebound of G’ and G”[30].

### Water content analysis

**Figure 8.**
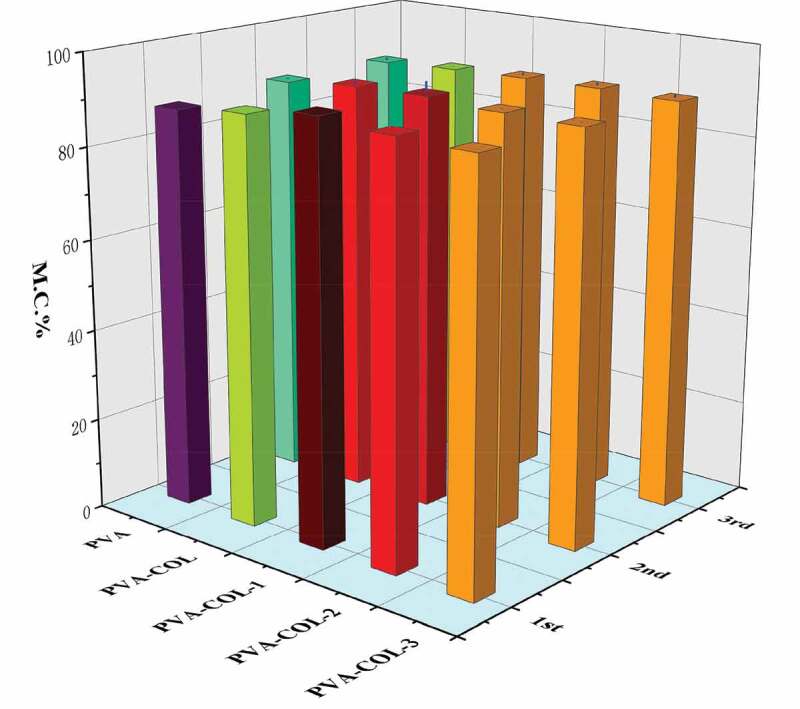
Water content of each group hydrogelin different freeze-thaw cycles

The water content of the hydrogel is closely related to the pores in the polymer network structure and the number of hydrophilic groups in the molecule. In addition, the crystallinity of PVA has a certain influence on the water content of the PVA hydrogel. The higher the crystallinity, the smaller the pores of the hydrogel and the weaker the ability to store water [31].It can be seen from Figure 8 that the introduction of COL has no significant effect on the water content of the hydrogel. With the increase of PGAD content in the hydrogel, the water content of PVA-COL-PGAD hydrogel first increased and then decreased. The reason for the increase is that PGAD, as a polymer material with water absorption properties, can form a certain three-dimensional network structure to lock moisture. The decrease in water content can be attributed to the entanglement of a large number of PGAD molecular chains, which makes the connection between the molecules closer, resulting in the reduction of pores in the internal network space structure of the hydrogel, resulting in a certain amount of water loss. In addition, the weaker degree of increase and decrease in water content reflects that the decrease in PVAcrystallinity has a weak effect on the water content of the hydrogel.

### Water return rate analysis

**Figure 9.**
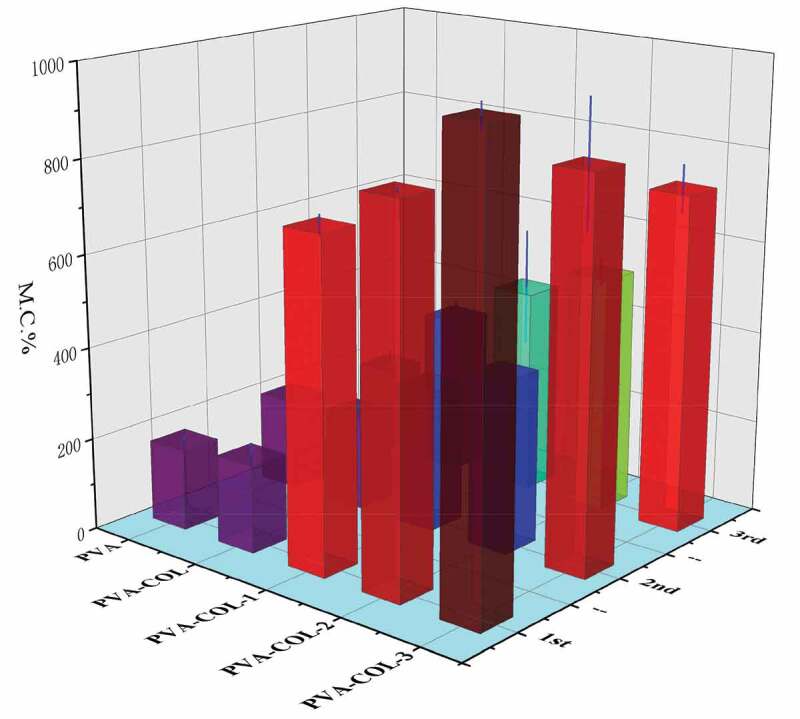
The swelling ratio of hydrogels in different freeze-thaw cycles

In the process of wound healing, it is usually faced with a situation where the volume of fluid discharged from the wound is small. In order to maintain the moist environment of the wound, it needs to be compensated by external moisture [32]. Therefore, we have studied and analyzed the swelling properties of the hydrogels of PVA, PVA-COL and PVA-COL-PGAD.The effects of PGAD content on the water absorption of the hydrogel were illustrated in Figure 9, which showed that the water absorption increased with the increase in the amount of PGAD.Previous studies have shown that in the continuous process of PVA hydrogel swelling, external water molecules will diffuse into the inside of the hydrogel over time, and the PVA molecular chain will gradually relax. After a period of time, the entire PVA molecular chain is fully stretched in water to make the hydrogel reach a swelling equilibrium. The introduction of COL does not significantly enhance the water absorption of the hydrogel, which is related to the lack of obvious interaction between COL and PGAD. The introduction of PGAD significantly enhances the water absorption of the hydrogel, which is mainly attributed to the fact that PGAD is a water-absorbing material and is closely related to the multi-structure caused by the introduction of PGAD.

### In vitro biocompatibility

**Figure 10.**
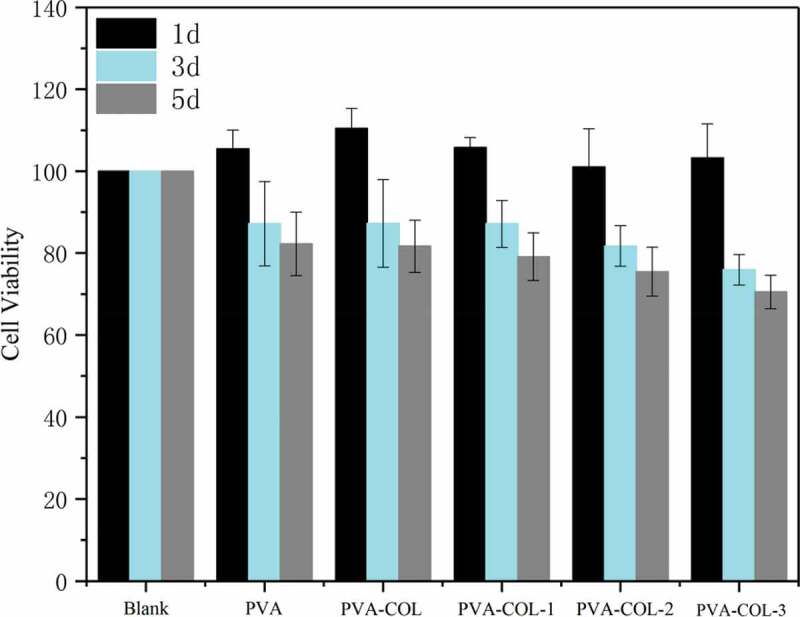
Cytotoxicity analysis of hydrogels

**Figure 11.**
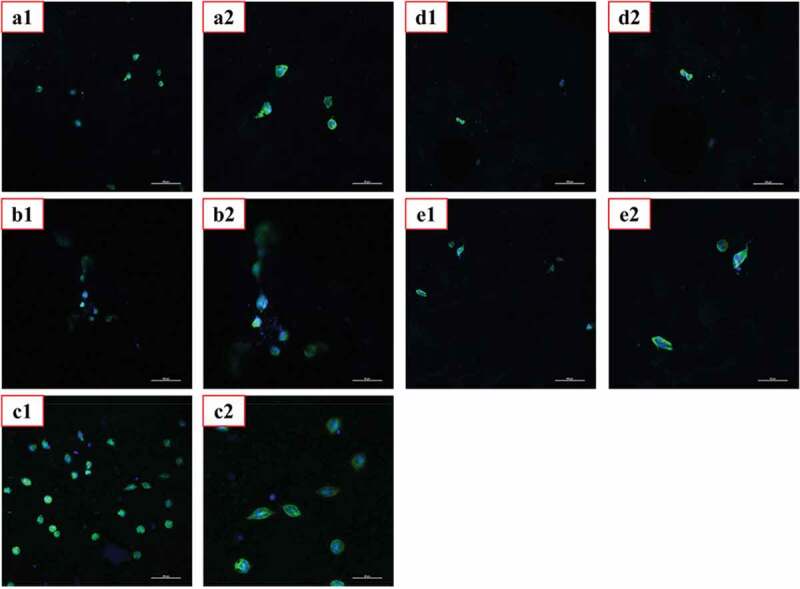
CLSM photographs of L929 fibroblasts cultured for 3 days on hydrogels.(a: PVA-COL, b: PVA-COL, c: PVA-COL-1, d: PVA-COL-2, e: PVA-COL-3)

Good biocompatibility is the most basic and most important performance of biomedical materials.We evaluated the cytotoxicity of the materials using MTT assay and CLSM, and the corresponding results were shown in Figures 10 and 11, respectively.As shown in Figure 10,the co-culture of L929 cells with the extracts of PVA, PVA-COL and PVA-COL-1 groups showed good proliferation, and the cytotoxicity was 0 to 1. Among them, PVA-COL extract has the highest cell proliferation rate. This is because PVA and COL have good cell compatibility, and a small amount of collagen in the extract can promote cell proliferation [33,34]. The cell proliferation rate in the extracts of PVA-COL-2 and PVA-COL-3 is low, which may be caused by the massive dissolution of the PGAD material during the extraction process, which leads to excessive concentration, which inhibits cell growth and proliferation.In addition, The observations by CLSM were in accordance with MTT assay, that the number of cells on the surface of the PVA-COL-1 hydrogel is the largest and the growth is good, indicating that the introduction of a certain amount of PGAD can promote cell proliferation and growth.

### In vivo biocompatibility

**Figure 12.**
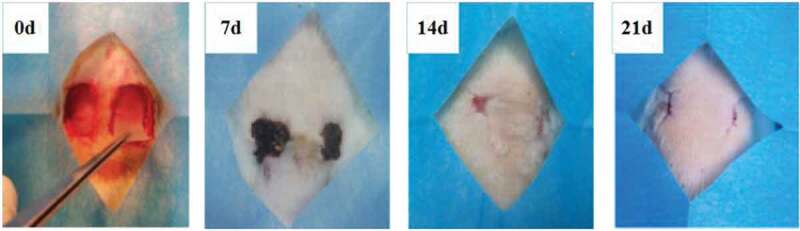
Photographic images showing healing pattern of wounds on different days (Left: Blank group, Right: PVA-COL-1)

**Figure 13.**
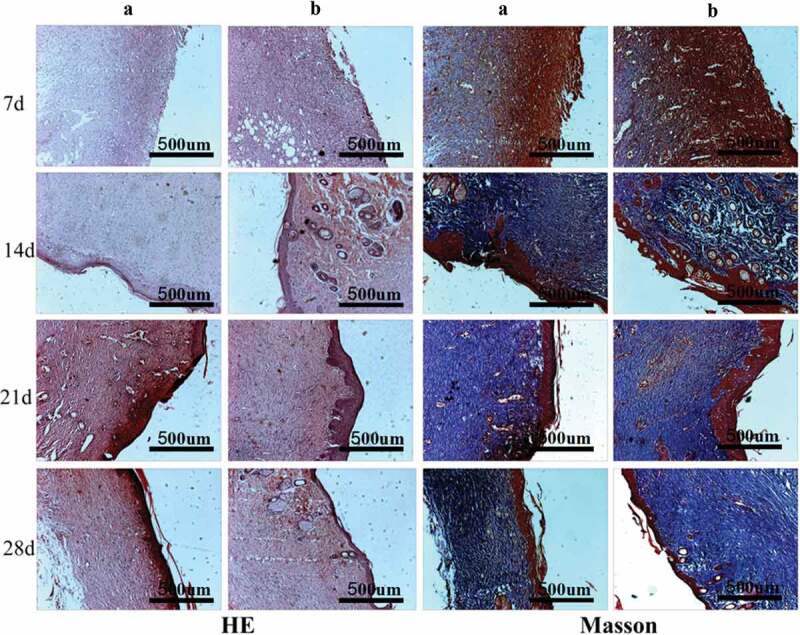
The HE/Masson staining of wound areas treated by PVA-COL-1 and blank group(A: Blank group B: PVA-COL-1. In HE staining, cytoplasm and extracellular matrix are red, chromatin in nucleus and cytoplasm are purple-blue; in Masson staining, muscle fibers, red blood cells, and cytoplasm are red, collagen fibers are blue, and nuclei are blue-black)

**Figure 14.**
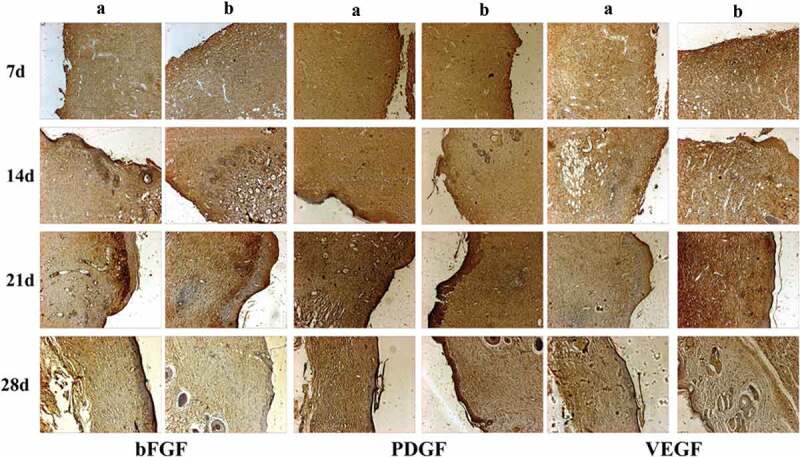
The bFGF, PDGF and VEGF immunohistochemical analysis of wound areas treated by PVA-COL-1 group and blank group(A: Blank group B: PVA-COL-1)

The in vivo biocompatibility of PVA-COL-1 was evaluated through its wound healing ability.As shown in Figure 12,the ability of PVA-COL-1(Figure 12, right) to heal wounded rats was compared with that of control(vaseline gauze; Figure 12, left). The wound on the right healed significantly faster than the wound on the left. Two weeks after the operation, the wound healing rate of PVA-COL-1 was greater than 70%, which was much higher than that of the control group. The wound healed completely after 3 weeks, and the moist environment is likely to promote wound healing. In addition, the PVA-COL-1 hydrogel introduced with PGAD is found to be hydrophilic and highly biocompatible, which may promote the migration of epidermal cells and induce skin recovery, thereby speeding up wound healing. Figure 13 shows the H&E stained wound images at different time points. Two weeks after the operation, the epidermis began to develop, the dermis was partially repaired, and the newly formed collagen fibers were loosely bent between each other [35].Three weeks after the operation, the epidermis formed at the wound and the thickness of the epidermal layer increased. The wound healing was basically completed. The thickness of the epidermis of the experimental group was significantly better than that of the blank group. Four weeks after the operation, both the experimental group and the blank group showed normal tissue structures such as hair follicles and small blood vessels, and the healing was basically completed.At the same time, it was discovered during the experiment that the wounds of the experimental group treated with hydrogel did not appear to adhere to the wounds. The reason is that the hydrogel can absorb wound exudate and keep the wound moist, while the control group only uses gauze. Although gauze can also quickly absorb wound exudate, it did not have good water-holding capacity, causing rapid water loss and adhesion. Experiments showed that the experimental group had a stronger effect on promoting wound healing than the blank group.The healing process of skin lesions is controlled by various growth factors necessary for cell growth and tissue regeneration. Fibroblast growth factor (bFGF), vascular endothelial growth factor (VEGF), and platelet-derived growth factor (PDGF) have been shown to promote cell proliferation, capillary formation, epidermal regeneration, and wound healing. Immunohistochemistry is a specific and sensitive tool for studying bFGF, VEGF, and PDGF expression levels using specific staining [36]. The results of immunohistochemistry are shown in Figure 14. The positive expression of bFGF, VEGF and PDGF in the experimental group was significantly higher than that in the control group. In addition, the expression area of the experimental group was also larger than that of the control group. These prove that PVA-COL-1 hydrogel can promote tissue repair, indicating its potential use as a biomaterial for skin wound healing.

## Conclusion

We explored the possibility of polyglutamic acid grafted with dopamine as a water-absorbing substance to improve the problem of insufficient water absorption of PVA-COL hydrogel. The results show that the introduction of PGAD can significantly enhance the water absorption capacity of the hydrogel, and at the same time can generate hydrogen bonds with PVA and COL to affect the crystallinity of the hydrogel. The in vitro biocompatibility evaluation shows that the introduction of a certain amount of PGAD is beneficial to the cytotoxicity of the hydrogel. In vivo cell compatibility experiments show that PVA-COL-1 can promote wound repair and speed up the healing process. In short, we have developed a hydrogel with good biocompatibility and physicochemical properties, which has the potential to be used in wound repair
